# Strike Fast, Strike Hard: The Red-Throated Caracara Exploits Absconding Behavior of Social Wasps during Nest Predation

**DOI:** 10.1371/journal.pone.0084114

**Published:** 2013-12-26

**Authors:** Sean McCann, Onour Moeri, Tanya Jones, Catherine Scott, Grigori Khaskin, Regine Gries, Sean O'Donnell, Gerhard Gries

**Affiliations:** 1 Department of Biological Sciences, Simon Fraser University, Burnaby, British Columbia, Canada; 2 Department of Biodiversity, Earth & Environmental Science, Drexel University, Philadelphia, Pennsylvania, United States of America; Universidade de São Paulo, Faculdade de Filosofia Ciências e Letras de Ribeirão Preto, Brazil

## Abstract

Red-throated Caracaras *Ibycter americanus* (Falconidae) are specialist predators of social wasps in the Neotropics. It had been proposed that these caracaras possess chemical repellents that allow them to take the brood of wasp nests without being attacked by worker wasps. To determine how caracaras exploit nests of social wasps and whether chemical repellents facilitate predation, we: (1) video recorded the birds attacking wasp nests; (2) analyzed surface extracts of the birds' faces, feet, and feathers for potential chemical repellents; and (3) inflicted mechanical damage on wasp nests to determine the defensive behavior of wasps in response to varying levels of disturbance. During caracara predation events, two species of large-bodied wasps mounted stinging attacks on caracaras, whereas three smaller-bodied wasp species did not. The “hit-and-run” predation tactic of caracaras when they attacked nests of large and aggressive wasps reduced the risk of getting stung. Our data reveal that the predation strategy of caracaras is based on mechanical disturbance of, and damage to, target wasp nests. Caracara attacks and severe experimental disturbance of nests invariably caused wasps to abscond (abandon their nests). Two compounds in caracara foot extracts [sulcatone and iridodial] elicited electrophysiological responses from wasp antennae, and were also present in defensive secretions of sympatric arboreal-nesting *Azteca* ants. These compounds appear not to be wasp repellents but to be acquired coincidentally by caracaras when they perch on trees inhabited with *Azteca* ants. We conclude that caracara predation success does not depend on wasp repellents but relies on the absconding response that is typical of swarm-founding polistine wasps. Our study highlights the potential importance of vertebrate predators in the ecology and evolution of social wasps.

## Introduction

It is well recognized that ants are important predators of social wasps, and that wasps exhibit ant-specific defensive adaptations [1]–[3], but until recently little attention has been paid to the role of vertebrate predators in social wasp evolution. Few vertebrate predators are known to specialize on the brood (larvae and pupae) of social wasps as their primary food source, but behaviors such as stinging, venom spraying [4], biting [5], [6], and physical fortification or camouflage of wasp nests [7]–[9] suggest selection for specific anti-vertebrate defensive tactics. Among vertebrate predators are birds such as the Honey Buzzards of the Old World [10]–[12] and the Red-throated Caracara, *Ibycter americanus*, of the New World [13]–[15]. The Red-throated Caracara (henceforth “caracara”) is unusual among the Falconidae in that it has well-developed cooperative breeding, with up to six adult individuals participating in brood care [13]. Caracaras are also known to forage in groups and to share large wasp nests [15]. In a previous study [13] we showed that wasp nests account for 59–77% of food items brought to caracara chicks, implying that caracara adults routinely engage in wasp nest predation.

**Figure 1:**
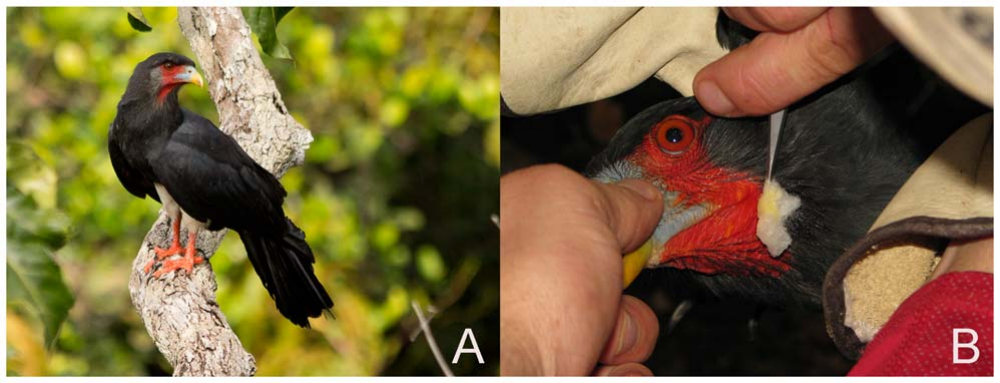
Photographs of Red-throated Caracaras. A. Red-throated Caracara perched on a branch near the Pararé Camp of the Nouragues Reserve in Central French Guiana, April 2011; note the bird's bare face and throat. B. Procedure of swabbing the skin of the bird's face with hexane-soaked cotton to remove skin surface chemicals. Feet and feathers were sampled in a similar fashion.

While Honey Buzzards have dense facial plumage and long narrow nares (nostrils) that presumably shield them from stinging wasps [16], these types of physical protections are not evident in caracaras, which lack plumage on the face and throat (Fig. 1 A, B). Instead, chemical rather than physical defenses have been posited to protect caracaras from attacking wasps. Thiollay [15], studying caracaras in French Guiana, observes: “The most striking adaptation of the Red-throated Caracaras was their ability to repel totally even the strongest and most aggressive wasps. As soon as one bird reached a nest, all the insects abandoned it and never attacked the raider, nor followed it when it carried the nest away. The wasps flew at a distance around the bird, rarely coming nearer than 1 m as long as it was on the nest.” Thiollay concludes: “The fact that wasps never attacked, nor even closely approached the caracaras raiding their nests suggests the involvement of some powerful chemical repellent. This repelling power is known to the local Indians and hunters, who readily eat raptors but who do not consume the caracaras because of their reportedly strong smell and taste.”. Thiollay's intriguing hypothesis has some antecedents. During a collection trip to Mexico, Lowery and Dalquest [17] relate that caracaras were considered by the local indigenous people to have a toxic substance on their feathers, and Weldon and Rappole [18] report malodorous qualities of caracaras.

Chemical defenses in birds have rarely been documented. The Pitohui (*Pitohui dichrous*) is known as toxic to indigenous people of New Guinea [19] and possesses homobatrachotoxin as a potent anti-predator poison. The Green Woodhoopoe (*Phoeniculus purpureus*) is said to have a foul-smelling secretion that deters predators [20]. Uropygial gland secretions of the Crested Auklet (*Aethia cristata*) are implicated as chemical protectants against ectoparasites [21], [22], whereas some shearwaters (Procellariidae) eject stomach oils that they use to repel or even kill avian attackers [23].

Most species of social wasps preyed upon by caracaras are swarm-founding members of the tribe Epiponini that abandon their nests in response to strong physical disturbance [24]. Triggering this absconding response of swarm-founding wasps may allow caracaras to prey on wasp nests without being severely stung. It is also possible that caracaras are immune to the venom of their prey, and simply withstand the stinging defense.

Here we tested the hypothesis that caracaras possess a chemical repellent that protects them from wasp attacks, and the alternate hypothesis that caracaras inflict severe mechanical damage on wasp nests and then rely on the absconding response of wasps. To test these hypotheses and to gather data on how caracaras attack nests of social wasps, we took three approaches: (1) we built a recording arena with four video cameras, supplied the arena with active wasp nests, lured in caracaras by playback of their territorial calls, and video recorded caracaras attacking the nests (2) we captured caracaras, took solvent-soaked cotton swabs of their faces, feet and feathers, and analyzed swab extracts for potential defensive or repellent chemicals; and (3) we inflicted mechanical damage on wasp nests to determine whether it causes wasps to abscond without stinging.

## Methods

### Study site

We conducted our fieldwork at the Inselberg and Pararé camps of the Nouragues Reserve in Central French Guiana (100 km SSW of Cayenne, 4°05′ N–52°41′W), an undisturbed lowland rainforest (55–410 m ASL) closed to most human activity for approximately 40 years [25]. This area has year-round populations of caracaras and a trail network to facilitate access to the forest [15].

### Observations of wasp nest predation by caracaras

In each of four field seasons (2008–2011) totaling 11 months, we observed caracara predation on wasp nests while we were on regular walks through the forest. During observations of wasp nest predation we attempted to identify adult wasps by visual inspection or by collection of brood or callow workers from nests.

### Controlled recordings of wasp nest predation by caracaras

**Figure 2:**
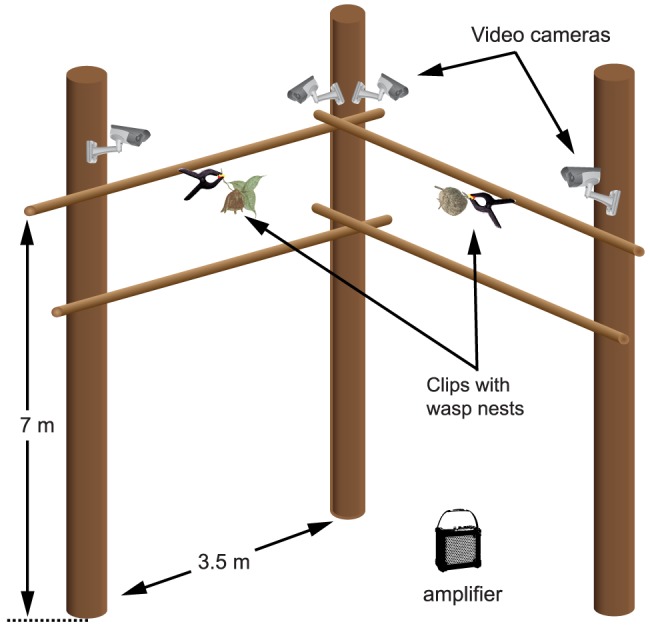
Drawing of the recording arena. We designed and constructed this arena to video record attacks of Red-throated Caracaras on active wasp nests which we transferred at night (when aggressive wasps are docile) to the arena from surrounding forest.

In 2010 and 2011, we recorded caracara predation on wasp nests in a recording arena (Fig. 2) constructed about 100 m northeast of the Inselberg Camp. We fitted the arena with four 540 TV line resolution security video cameras (Aartech Canada, Oshawa ON, Canada) and fed video signals to a 4-channel security digital video recorder (Channel Vision DVR 4C, Channel Vision Technology Costa Mesa, CA, USA) housed in a shelter within the camp. At night, when the otherwise aggressive wasps are docile and remain on their nests, we transferred active wasp nests from the surrounding forest to the arena. We used a ladder to access the arena and attached the nests with spring clips to crosspieces 7 m above ground (Fig. 2).

The arena was designed to record two nests concurrently. Upon motion detection, the DVR was set to record video at either 640×288 pixel resolution at 24 frames per second (FPS), or at 640×480 resolution at 15 FPS, depending on the number of cameras employed. A 5-s pre-recording buffer ensured that the entire sequence of events prior to motion detection was recorded. This pre-recording buffer transfers the footage 5 s prior to motion detection to the hard drive, a technology commonly found on security DVRs.

We played back caracara calls using a Roland Edirol R-09 portable field recorder and a Roland Microcube amplifier (Roland Corporation, Los Angeles, CA, USA) to attract birds to the camp, and then switched the playback device to a second amplifier located immediately below the arena.

We recorded 11 attacks by caracaras on nests of five wasp species (four nests each of *Polybia bistriata* and *P. jurinei*, and one nest each of *P. scrobalis*, *P. belemensis* and *P. affinis*). These nests were attacked within 2–27 days after transplantation. We selected *Polybia* nests for recordings because they were most frequently brought as prey to a caracara nestling in two seasons of provisioning observations [13]. Also, *Polybia* wasp species represent a wide range of body size and aggressiveness, and are diverse and widespread across the geographic distribution of caracaras.

For each attack, we viewed the video to determine whether caracaras were attacked and possibly stung by wasps, as evidenced by wasps flying towards a caracara to intercept the bird, or by caracaras scratching or plucking wasps off their faces and feathers. We also calculated the time it took caracaras to complete the attack, defined as the period within which a caracara first appeared perched in the field of view of one of the cameras until it tore into the wasp nest with its beak, or knocked the nest off the plant substrate. If caracaras were apparently driven from the nest area by the wasps, we recorded the time elapsed until the birds returned. We recorded an “absconding response of wasps” when all wasps departed from the nest envelope.

We calculated the Spearman's Rank Correlation Coefficient as a measure of correlation between the size of wasp nests and the time caracaras required to complete nest attacks [26]. We used a Wilcoxon Rank-Sum test to compare the time for completion of attacks between nests that were defended and those that were not. We performed all tests in R 2.15.2.

### Acquisition of potential repellents from the faces, feathers or feet of caracaras

We captured five caracaras in the forest surrounding the Inselberg camp by luring them into a mist net with a hand-carved conspecific decoy and playback of territorial calls [13]. The permits for the attraction and capture procedure were approved by the Animal Care and Use Committee of Simon Fraser University (Protocol number 849B-07) as well as the Direction Régionale de l'Environnement de Guyane (DIREN), and all permits complied with all relevant regulations. We took great care to avoid injury to birds, hooded them to minimize stress, marked them with colored Darvic plastic bands, and released them unharmed. We smelled each bird and noted any unusual or unpleasant odors. Following standard procedures [27], we surface-extracted the bare skin of each bird's face and neck, the scaly skin of its feet and tarsi, and its contour feathers on the back and breast, using in sequence cotton swabs soaked in distilled hexane or methanol to extract chemicals of potentially different polarity. Swabs of the face and throat typically left a yellowish stain on the cotton (Fig. 1 B). We then placed the cotton swabs in glass vials, added 200 µl of solvent, and kept samples at −4°C until they could be analyzed in the laboratory.

### Gas chromatographic-electroantennographic detection analysis of cotton swab extracts of the caracaras' faces, feathers or feet

We anticipated that any defense chemicals of caracaras repellent to sympatric prey wasps would need to be perceptible to wasps and thus would elicit antennal responses which then could help determine the key components for chemical identification. Therefore, we collected adult wasps from two *P. occidentalis* nests (4°52′44″N, 52°20′06″W) and used their antennae in gas chromatographic-electroantennographic detection (GC-EAD) analysis [28], [29] of combined cotton swab extracts from the caracaras' faces, feathers and feet. *Polybia occidentalis* is a representative prey species with broad Neotropical distribution and a high degree of sympatry with caracaras. For GC-EAD analyses, we used a Hewlett-Packard (HP) 5890 gas chromatograph fitted with a GC column (30 m×0.25 mm ID) coated with DB-5MS (J&W Scientific, Folsom, California, USA). For each recording (n = 15), we removed an antenna from a wasp's head, and suspended it between two glass capillary electrodes (each 1.0×0.58×100 mm; OD×ID× length; A-M Systems, Inc., Carlsborg, Washington, USA) filled with saline solution [30]. We further analyzed compounds that elicited consistent antennal responses by GC-mass spectrometry (MS) on a Varian Saturn 2000 Ion Trap GC-MS fitted with the DB-5MS column, using separate hexane extracts of the caracaras' face, feathers and feet. The temperature program for GC-EAD and GC-MS analyses was 50°C (for 3 min), 20°C per min to 280° (held for 5 min).

### Collection and analyses of defensive secretions from *Azteca* ants

Three compounds in caracara foot extracts [2-heptanone, sulcatone, an iridodial isomer] (see Results) are also known to occur in defensive secretions of dolichoderine ants, including *Tapinoma* spp. [31] and *Azteca* spp. [32]. *Azteca chartifex* is abundant at our study site, which led us to predict that caracaras coincidentally acquire chemicals from *A. chartifex* or other dolichoderine ants when they alight on ant-inhabited trees. To compare chemicals present in *Azteca* ants with those present on caracaras, we located (near the Pararé Camp) the large carton nests of *Azteca* nr. *chartifex* (more specific taxonomic determination was not possible based on our collections of worker ants), placed glass capillary tubes (1.5×100 mm) into the terminal end of these nests, extracted defensive secretions from the tubes with hexane, and stored these extracts at −4°C prior to GC-MS analysis as described above.

### Physical disturbance of wasp nests

To determine whether physical damage, as might be inflicted by caracaras, would trigger a stinging defense or an absconding response of wasps, we conducted the following four manipulations, in sequence, on ten nests of *P. bistriata*. We (1) tapped the nest substrate three times while grasping the nest base with a gloved hand; (2) stroked the nest three times with a gloved hand while grasping the nest substrate; (3) tore the nest envelope with a sharp object while grasping the nest base with a gloved hand; and (4) tore the nest from the plant substrate, placed it on the ground, and tapped it with a finger. We waited two days between applications of each of the four treatments to an individual nest. In each replicate of each treatment, we counted the number of wasps attempting to sting. The final disturbance was replicated only 9 times, as the tenth nest had been raided by ants in the interim. The average nest size was 5.9±1.3 cm (mean ±SD) at its widest point, and each nest contained capped brood (as determined after nest destruction).

## Results

### Field observations of wasp-nest predation by caracaras

During 11 months of field work, we witnessed attacks by caracaras on two wasp nests. In most instances when we approached, the birds ceased foraging, alarm-called, and flew off. However, on 28 January 2008, we observed a group of five caracaras feeding on the brood of a large *Polybia dimidiata* nest (∼50 cm diameter) located 20 m above ground. The nest had large holes in the upper and lower envelope. As many as three birds were perched on the nest at a time. While we observed the event for 36 min, and filmed it for 20 min, a large number of wasps flew around the birds at a distance of several meters, but no wasps approached the birds (Video S1). We made a second observation of a single caracara feeding on a small (10 cm diam.) *Polybia* nest, but we did not witness the commencement of this attack, nor were we able to collect workers for identification.

### Controlled recordings of wasp nest predation by caracaras

Caracaras successfully attacked all nests of *P. bistriata*, *P. belemensis* and *P. scrobalis* (Table 1). In no instance did the wasps mount a detectable defense, but instead flew away upon nest disturbance. Caracaras ate the brood of all small nests but one *in situ*. In the exceptional event, a color-banded female attacked a *P. bistriata* nest, plucked it with her beak from the branch, and flew off with it.

**Table 1:** Summary of video-recorded observations of caracara attacks on nests of various species of *Polybia* wasps.

Wasp species	Mean wing length (mm) of workers^1^	Diameter (cm) of nest	Number of caracaras present	Mode of caracara attack	Caracara counterattacked by wasps?	Time (s) to complete attack^2^	Date	Video
*P. affinis*	12.0	6.6	1	Brood eaten, in situ	yes	172	21-Feb-11	S2
*P. belemensis*	6.27	13	2	Brood eaten *in situ*	no	89	21-Apr-10	n/a
*P. bistriata*	6.85	5.2	2	Nest plucked and taken away	no	64	16-Mar-10	S3
*P. bistriata*	6.85	5.6	3	Brood eaten *in situ*	no	39	2-Apr-10	n/a
*P. bistriata*	6.85	6.2	2	Brood eaten *in situ*	no	51	21-Apr-10	S4
*P. bistriata*	6.85	5.8	2	Brood eaten *in situ*	no	58	23-Apr-10	S5
*P. jurinei*	9.2	12	2	Nest struck to the ground	yes	257	26-Mar-10	S6
*P. jurinei*	9.2	11	3	Brood eaten *in situ*	yes	196	2-Apr-10	S7
*P. jurinei*	9.2	12	3	Nest struck to the ground	yes	608	26-Mar-11	S8
*P. jurinei*	9.2	15	2	Nest struck repeatedly, brood eaten *in situ*	yes	1241	23-Apr-11	S9
*P. scrobalis*	7.07–7.57	8	1	Brood eaten *in situ*	no	87	21-Feb-11	S10

^1^ All data on wasp size except for those of *P. bistriata* from Richards [47]; data on *P. bistriata* from Richards and Richards [48].

^2^ Period within which a caracara first appeared in the field of view of one of the cameras until it tore into the wasp nest with its beak or knocked the nest from the substrate.

In two of the attacks on *P. bistriata* nests, the caracaras repeatedly pulled on the nest substrate, dislodging or driving most of the wasps off in the process. As soon as the caracaras disturbed the nest or tore into the envelope, the remaining wasps departed (Video S3, S4).

During the attack on the *P. affinis* nest, the caracara first landed beside the nest, coming under attack by wasps flying off the envelope. The bird departed, then returned and approached on the upper crosspiece and had a wasp attack its face on the left side. The bird scratched off the wasp with its left foot, and henceforth experienced no further counterattacks, not even when it was tearing into the nest and consuming the brood (Video S2).

**Figure 3:**
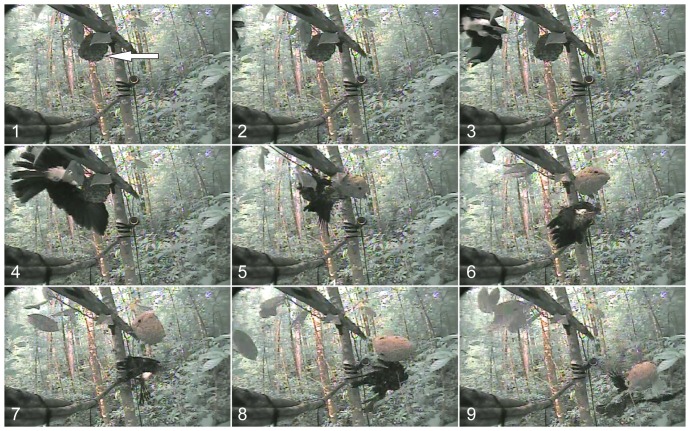
Fly-by attack of Red-throated Caracara on nest of *Polybia jurinei*. Single-frame images of a video recording revealing the fly-by attack of a Red-throated Caracara (ventral view) on a large nest of *Polybia jurinei* (arrow in panel 1) that we had transferred to the recording arena (see Figure 2). In the corresponding video S6, it is apparent that the wasps take off from the nest envelope to intercept the bird approaching from the left side (panels 1–3). Note that the nest is dislodged by the bird's talons (panel 4).

In contrast to *P. bistriata*, caracaras suffered counterattacks from defending wasps during all attacks on *P. jurinei* nests. These included four instances of wasps flying from their nest to intercept a caracara approaching in flight (e.g. Video S7). During one of the caracara attacks, the wasps' counterattack was so fierce that it prompted the birds to temporarily retreat four times (Video S6), although the birds usually returned within 10–90 seconds (Table 2). During two predation events, caracaras mounted rapid fly-by attacks on the nest, striking it with their talons and eventually causing the nest to fall to the ground (Videos S6, S8, Fig. 3) where the birds consumed the brood. In the attack on the smallest *P. jurinei* nest (Video S7), a caracara was counterattacked both in flight and after alighting near the nest, prompting the bird to scratch and pluck wasps from its plumage. However, when two caracaras began tearing into the nest envelope, the wasps absconded.

**Table 2:** Number of instances where caracaras were driven away by attacking *Polybia* workers.

Wasp species	Number of times caracara(s) were driven away	Time (s) to return of caracara(s)	Date	Video
*P. scrobalis*	1	46	21-Feb-11	S10
*P. jurinei*	3	40, 20, 98	26-Mar-10	S6
*P. jurinei*	1	10	2-Apr-10	n/a
*P. jurinei*	2	51, 11	26-Feb-11	S7
*P. jurinei*	1	960	23-Apr-11	S9

In the attack on the largest *P. jurinei* nest, a single caracara flew low over the nest, and while passing was attacked by several wasps. After this, the caracara struck the crosspiece holding the nest four times, and the nest directly a single time, before a caracara perched next to the nest and began tearing into it with its beak. At this point, the remaining wasps absconded, leaving the caracara to eat the brood *in situ* (Video S9).

The two species of wasps (*P. jurinei*, *P. affinis*) that defended their nests against caracara attacks have workers that on average are larger than the workers of those species (*P. belemensis*, *P. bistriata*, *P. scrobalis*) that offered no defense (Table 1, Fig. S1). Furthermore, caracaras took longer to complete attacks on species that did defend their nest (median 257 s, range 172–1240 s) than on those that did not (median 61 s, range 39–89 s; Wilcoxon Rank-Sum Statistic: 30, n = 5, m = 6, *p*<0.01 (Table 1). There was also a strong positive correlation between the time taken to complete attacks and the diameter of nests (Spearman's ρ = 0.82, *p*<0.01, Table 1, Figure S1).

### Gas chromatographic-electroantennographic detection (GC-EAD) analysis of cotton swab extracts of the caracaras' faces, feathers and feet

**Figure 4:**
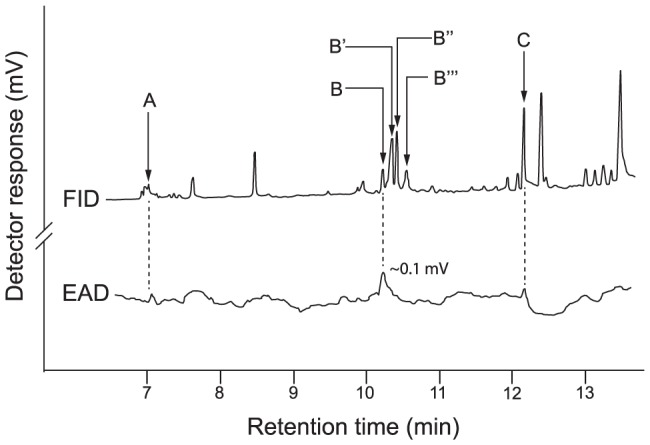
Gas chromatographic-electroantennographic detection analysis of combined cotton swab extracts of a caracara's face, feathers and feet. Representative recording (n = 15) of flame ionization detector (FID) and electroantennographic detector [EAD: antenna of a female wasp of *Polybia occidentalis*] responses to aliquots of combined cotton swab extracts of a Red-throated Caracara's face, feathers and feet. 6-Methyl-5-hepten-2-one (A), an epimer of *cis, trans*-iridodial (B), and tetradecanoic acid (C) elicited consistent antennal responses. B′, B″ and B’’’ refer to other iridodial isomers. Chromatography: splitless injection, injector and detector temperature: 240°C; DB5-MS column; temperature program: 50°C (3 min), 20°C per min to 320°C; see methods for details.

We captured caracaras 16 times in the course of our fieldwork, and none of the birds we captured had unpleasant odors during handling. Because chemical constituents in hexane and methanol extracts were similar, we present analytical results pertaining to only the hexane extract. GC-EAD and GC-MS analyses of combined cotton swab extracts of the birds' faces, feathers or feet revealed three compounds that consistently elicited antennal responses from wasps: 6-methyl-5-hepten-2-one (sulcatone), an epimer of *cis, trans*-iridodial, and myristic acid (Fig. 4, see Document S1 for detailed analytical and synthetic descriptions). Although present in cotton swab extracts, other iridodial isomers did not elicit antennal responses. GC-MS analyses of separate face, feather, and feet extracts revealed that sulcatone, 2-heptanone and iridodial were present in foot extracts but not face or feather extracts.

### Collection and analyses of defensive secretions from *Azteca* ants

**Figure 5:**
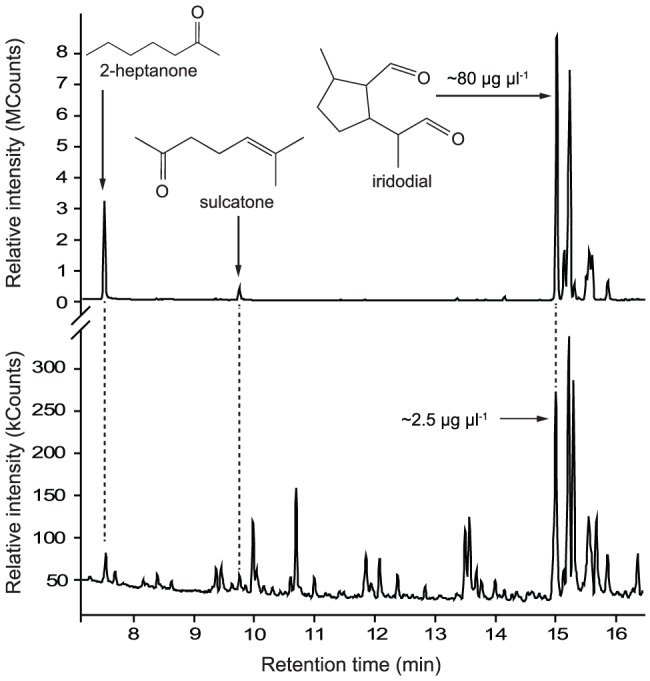
Comparison of chemicals from the Red-throated Caracara and *Azteca* nr. c*hartifex* ants. Total ion chromatograms of aliquots of a defensive secretion extract of *Azteca* nr. *chartifex* ants (top) and a cotton swab foot extract of Red-throated Caracaras (bottom). Dashed lines refer to compounds present in both samples. Note the occurrence of 2-heptanone, sulcatone and an epimer of *cis, trans*-iridodial in both extracts. Chromatography: Varian 3800/Saturn 2000 Ion Trap GC-MS; splitless injection; injector temperature 240°C; DB5-MS column; temperature program: 50°C (3 min.) 20°C per min. to 280°C; see methods for details.

When we disturbed *Azteca* nests by inserting a glass capillary tube in the nest carton, many ants emerged and attacked the capillary tube by biting and secreting pygidial gland content. This defensive secretion appeared as a white, sticky fluid (Video S11). GC-MS analyses of these gland secretions revealed 2-heptanone, sulcatone and several isomers of iridodial. The two ketones and the same isomers of iridodial were also present in caracara foot extracts (Fig. 5), but not in the face or feather extracts.

### Physical disturbance of *P. bistriata* nests

No physical mode of nest disturbance [tapping (1), stroking (2), envelope tearing (3), and placement on the ground (4)] elicited stinging responses from wasps. During modes 1–3, the wasps flew about and settled on nearby vegetation but did not attempt to sting. Subsequent to disturbance modes 1–3, the workers returned to their nests after several minutes. In contrast, each nest we detached from the substrate and placed on the ground was abandoned within seconds (Table 3), and the workers did not return.

**Table 3:** Stinging and evacuation behavior by worker wasps of *Polybia bistriata* in response to various types of disturbance of their nest. Absconding is defined as all wasps leaving the nest and not returning.

Type of disturbance	n	Wasps attempting to sting	Evacuation
Tapping	10	0	Partial
Stroking	10	0	Partial
Tearing	10	0	Partial
Knocking down nest	9	0	Absconding

## Discussion

Our data support the following conclusions: (1) caracaras engage in a “hit-and-run” predation tactic when they attack nests of highly aggressive and defensive wasps; (2) the resulting nest damage prompts wasps to abscond; (3) the caracaras' tactic of forcible physical attack and flight reduces the risk of getting stung; (4) there are no detectable repellent chemicals on faces and feathers of caracaras; and (5) the chemicals on the caracaras' feet likely originate from *Azteca* ants, but do not seem to fend off attacking wasps.

Our video recordings of wasp nest predation by caracaras did not support the hypothesis of a chemical defense that protects caracaras from counterattacking social wasps. On the contrary, caracaras were vigorously attacked (even in flight) and likely stung by two of the wasp species we studied. The caracaras avoid excessive wasp attacks by inducing absconding of the colony before they feed on the brood or carry the nest away.

When a caracara attacked, small-bodied workers of *P. bistriata* nests did not attempt to sting the bird, as they did not attempt to sting us when we experimentally disturbed their nests. Facing a determined and persistent attacker, *P. bistriata* does not attempt to defend. However, large wasp species, such as *P. jurinei*, do attempt to sting the attacking bird for some time, but given sufficient disturbance of the nest they will also abscond. The specific defense behavior exhibited by each wasp species likely reflects their ability, or inability, to repel large avian predators, and may be related to relative sting potency and worker numbers. Previous research has shown that small-bodied wasp species are generally less likely to defend than large-bodied species, and nests with a large brood are more predisposed to defend than nests with little brood [33]. Unsurprisingly, it took caracaras longer to complete an attack when the wasp nest was defended, although ultimately they destroyed all the nests.

The lack of stinging attacks by *P. bistriata* during experimental physical disturbance surprised us, because during unrelated activities this species did sting us several times when we accidentally brushed against a nest. The wasps' stinging defense in response to a slight nest disturbance may warn intruders to stay away from the nest, whereas intense disturbance may signal that attack by a determined predator is well under way and that defense is futile. In any case, *P. bistriata* workers responded similarly to disturbance by caracaras and humans.

In birds, the decision of parents to defend brood depends on their investment in the brood and their ability to drive a predator away [34]. Similarly, wasps may adjust defensive tactics depending on the predator they face. By abandoning a nest in response to the determined attack of a caracara, reproductive female and worker wasps preserve their potential to reproduce in the future, while sacrificing their current investment in eggs, brood, and nest materials. Other severe nest disturbances such as those caused by tree falls and army ant attacks also induce absconding in swarm-founding polistines [24], [35], [36], suggesting that coordinated abandonment of nests is a tactic that may minimize losses of worker wasps when a nest is faced with annihilation.

Larger wasp species, such as *P. jurinei*, seem to be capable of temporarily driving the caracaras away. Although the caracaras' fly-by attacks may reduce the probability of getting stung, it is conceivable that caracaras are stung repeatedly by counterattacking wasps. Excessive wasp or bee stings can be dangerous to many animals [37], and may have killed a Crested Honey Buzzard (*Pernis ptilorhynchus*) when it was counterattacked by honeybees [38]. Wasp venom resistance analogous to the snake-venom resistance of didelphid marsupials [39] could offer an alternative means of protection to caracaras, but this is yet to be studied.

The hit-and-run predation tactic of caracaras when they attack large wasp nests resembles the recently-documented behavior of Oriental Honey Buzzards attacking hornet nests [40]. The Honey Buzzards work in groups and apparently take turns in striking a hornet nest with their bodies and feet during fly-by attacks. Caracaras also cooperate during foraging [15]. In two of our videos documenting attacks on *P. jurinei* nests, two birds participated (Table 2). In one case, two birds tore into the envelope, in the other each of two birds struck the nest with their talons. We also documented several birds tearing into and sharing the brood of a large *P. dimidiata* nest. Group-living and cooperative foraging may be strategies that help caracaras share the risks and rewards of attacking formidable prey, such as *Synoeca* spp. [14], [41] and *P. dimidiata* [this study]. The advantages of group foraging in other species include minimizing the variation in daily success among cooperating individuals [42], which is important if prey is patchily-distributed and difficult to find.

The vulnerability of wasp nests to caracara attacks sheds light on other defensive adaptations of social wasps against vertebrate predators. Many social wasps in the Neotropics have visually cryptic nests, which likely reduce the rate of detection by diurnal vertebrate predators such as caracaras [9], [43]. Furthermore, many nests are located in dense tangles of branches and vines which may not only reduce detection by avian predators but also render rapid fly-by attacks difficult or impossible. Aggregated nesting, as the aggressive *P. rejecta* does in some locations [44], may allow several colonies to pool defenses against caracara predation. Such a tactic has been reported for some Asian honeybees defending against Bee-eaters (Meropidae) [45]. Finally, the massive mud nest envelopes of *Polybia* subgenus *Pedotheca* wasps (e.g. *P. singularis*) [7], [8], and the tough felt-like nests of *Chartergus* and *Epipona* wasps [46]–[48], may make it difficult for avian predators to inflict critical nest damage or to dislodge nests from branches. Such nests may render the caracaras' hit-and-run tactic impossible, although further studies would be needed to support this hypothesis.

The predation behavior of caracaras causes disturbance and damage to targeted wasp nests and induces absconding of worker wasps. This type of tactic does not necessarily require a strong chemical repellent to protect the birds from wasps [15], as absconding wasp colonies cease defensive stinging. The hypothesis of a chemical repellant was likely formulated [15] because the initial stage of the caracara attack and the absconding response of the wasps were not witnessed closely.

The types of compounds [iridodial, sulcatone, 2-heptanone, myristic acid] we found in foot samples of caracaras are not likely to fend off rapidly-flying wasps, and in fact some species of wasp build their nests in close association with ants that secrete iridodial, sulcatone and 2-heptanone for defense [32], [49]. The co-occurrence of these compounds on the feet of caracaras and in defensive secretions of *Azteca* ants implies that these compounds are ant-derived. Caracaras may acquire these compounds coincidentally while perching on trees inhabited with *Azteca* ants that vigorously attack animals on their trees, and/or while preying on *P. rejecta*, which is commonly commensal with *Azteca* ants [44].

Alternatively, caracaras may intentionally seek *Azteca* ants to anoint their feathers with ant secretions for protection from ectoparasites. This “anting” behavior has been reported in one other Neotropical falconid [50], but in that case the ant was an ecitonine, not a dolichoderine. Other anting birds, though, have been reported to seek iridodial-secreting ants [51]. However, because none of these ant-derived compounds was present in the feather samples of caracaras, intentional anting by caracaras probably cannot account for their presence. Nonetheless, the presence of these compounds on caracaras highlights a surprising connection between seemingly unrelated members of a tropical forest community, mediated by commensalism between ants and wasps, and predation by birds on wasps.

In summary, the predation tactic of caracaras is based on severe disturbance and damage to target wasp nests and ultimately relies on the absconding response of swarm-founding wasps. The hit-and-run predation tactic of caracaras when they attack the large nests of highly aggressive wasps reduces the risk of getting stung by counterattacking wasps. Further studies should investigate whether caracaras have immune adaptations to cope with wasp venom as the birds seem to suffer some stings during attacks on large wasp nests. In turn, the effect of defensive adaptations of social wasps, such as aggregate nesting of *P. rejecta* [40], and physical nest fortification by *P. singularis*, *Epipona* spp., and *Chartergus* spp., on the predation success of vertebrate predators merits further investigation.

## Supporting Information

Video S1 **Red-throated caracaras consuming brood from a nest of** ***Polybia dimidiata*** **, 28 Jan. 2008.**
(MP4)

Video S2 **Red-throated Caracara attacking nest of** ***Polybia affinis*** **, 21 Feb. 2011.**
(MP4)

Video S3 **Red-throated Caracara attacking nest of** ***Polybia bistriata*** **, 16 March 2010.**
(MP4)

Video S4 **Red-throated Caracara attacking nest of** ***Polybia bistriata*** **, 21 April 2010.**
(MP4)

Video S5 **Red-throated Caracara attacking nest of** ***Polybia bistriata*** **, 23 April 2010.**
(MP4)

Video S6 **Red-throated Caracara attacking nest of** ***Polybia jurinei*** **, 26 March 2010.**
(MP4)

Video S7 **Red-throated Caracaras attacking nest of** ***Polybia jurinei*** **, 2 April 2010.**
(MP4)

Video S8 **Red-throated Caracaras attacking nest of** ***Polybia jurinei*** **, 26 March 2011.**
(MP4)

Video S9 **Red-throated Caracara attacking nest of** ***Polybia jurinei*** **, 23 April 2011.**
(MP4)

Video S10 **Red-throated Caracara attacking nest of** ***Polybia scrobalis*** **, 21 February 2011.**
(MP4)

Video S11 **Collection of defensive secretion from** ***Azteca*** **NR** ***chartifex*** **workers, 18 November 2012.**
(MP4)

Document S1 **Detailed analytical and synthetic procedures used in determination of iridodial and other chemicals recovered from caracara foot-swab extracts.**
(DOCX)

Figure S1 **Time taken for caracaras to complete attacks on wasp nests depends on nest size and wasp defensive behavior.**
(EPS)
