# Supplementary material for: Strike Fast, Strike Hard: The Red-Throated Caracara Exploits Absconding Behavior of Social Wasps during Nest Predation
Source: PLoS One. 2013 Dec 26;8(12):e84114. doi: 10.1371/journal.pone.0084114 (PMC3873407; doi:10.1371/journal.pone.0084114)
Supplement: Document S1 — Detailed analytical and synthetic procedures used in determination of iridodial and other chemicals recovered from caracara foot-swab extracts. (DOCX) [file pone.0084114.s012.docx]

**S12: chemical analyses**

***Identification of compounds in cotton swab extracts of caracara feet that elicited responses from wasp antennae***

We identified the three compounds that elicited responses from wasp antennae in cotton swab extracts of caracara feet (A, B, and C in Fig. 4) as 6-methyl-5-hepten-2-one (sulcatone) (A), one or two epimers of *cis, trans*-iridodial (B), and tetradecanoic acid (C). Compound A had identical GC retention and mass spectrometric characteristics as an authentic standard (sulcatone) purchased from Sigma-Aldrich. Compound C had identical retention and mass spectrometric characteristics as synthetic tetradecanoic acid we prepared by oxidation of tetradecanol. The mass spectrum of compound B resembled that of nepetalactol or an iridodial (the corresponding di-aldehyde) [29]. The three compounds eluting immediately after compound B (B’, B” and B”’ in Figure 4) had a similar mass spectrum as B, suggesting that they are isomers of B.

Isomers of iridodial are abundant constituents in secretions of the ant *Tapinoma nigerrimum* [29], and on a BPX5 column (equivalent to a DB-5 column) elute in the following order: *cis*, *trans*, *trans*, *trans* (2 epimers), and *trans*, *cis* (2 epimers) [1,2]. As the *cis*, *trans*-iridodial eluted first, and the EAD-active compound B in (Fig. 4) was the first eluting isomer, we hypothesized that B is one or both epimers of *cis*, *trans*-iridodial.

To confirm this structural assignment*,* we initiated synthesis taking into account that *cis*, *trans*-nepetalactol and corresponding iridodials can be obtained from nepetalactone [2,3][51,52]. We purchased the essential oil of *Nepeta cataria* (Liberty Natural products Inc., Portland, Oregon, USA) which contains *cis*, *trans*- and *trans*, *cis*-nepetalactones in a 2.25:1 ratio by GCMS, similar to other reports of similar material [4]. We purified the nepetalactones in the essential oil by flash chromatography [pentane (95) : ether (5)] and treated them with 1,8-diaza-bicyclo(5.4.0)undec-7-ene in xylene, yielding the *cis*, *trans*-neptalactone as a single isomer. We then reduced this isomer with diisobutylaluminium hydride to afford *cis*, *trans*-nepetalactol as the major product. The reaction mixture also contained two minor products (~5%) which we assigned to be the corresponding iridodials of *cis*, *trans*-nepetalactol based on their mass spectra and a previous study [3] reporting that *cis*, *trans*-nepetalactol contained 5-8% of the corresponding iridodials (1*R*,2*S*,5*R*,8*R*-iridodial and 1*R*,2*S*,5*R*,8*S*-iridodial), which form as part of a chemical equilibrium. The first of two iridodials coeluted with B and had an identical mass spectrum. Based on these analyses, we conclude that EAD-active B in Figure 4 is a one or both epimers of *cis*, *trans*-iridodial.

**Literature Cited**

1. Oldham NJ (1994) Chemical studies on exocrine gland secretions and pheromones of some social insects. PhD thesis. Keele University, Keele UK.

2. Liblikas I, Santangelo EM, Sandell J, Baeckström P, Svensson M, et al. (2005) Simplified isolation procedure and interconversion of the diastereomers of nepetalactone and nepetalactol. J Nat Prod 68: 886–890. doi: <http://dx.doi.org/10.1021/np049647d>

3. Chauhan KR, Zhang Q-H, Aldrich JR (2004) Iridodials: enantiospecific synthesis and stereochemical assignment of the pheromone for the golden-eyed lacewing, *Chrysopa oculata*. Tetrahedron Lett 45: 3339–3340. doi: <http://dx.doi.org/10.1016%2Fj.tetlet.2004.03.034>

4. Wang M, Cheng K-W, Wu Q, Simon JE (2007) Quantification of nepetalactones in catnip (*Nepeta cataria* L.) by HPLC coupled with ultraviolet and mass spectrometric detection. Phytochem Anal 18: 157–160. doi: [http://dx.doi.org/10.1002/pca.965](http://dx.doi.org/10.1002/pca.965%20)
